# Evaluation of the move to remote delivery of drug and alcohol services during the COVID-19 pandemic: A study protocol

**DOI:** 10.1177/22799036221106583

**Published:** 2022-08-06

**Authors:** Nigel Lloyd, Wendy Wills, Suzanne Bartington, Charis Bontoft, Gavin Breslin, Olujoke Fakoya, Imogen Freethy, Jaime Garcia-Iglesias, Neil Howlett, Julia Jones, Katie Newby, Nigel Smeeton, Adam Wagner, Amander Wellings, David Wellsted, Katherine Brown

**Affiliations:** 1 University of Hertfordshire, Hatfield, UK; 2 University of Birmingham, Birmingham, UK; 3 Ulster University, Newtownabbey, UK; 4 University of East Anglia, Norwich, UK

**Keywords:** COVID-19, substance misuse, public health, addiction, treatment services

## Abstract

**Background:**

Substance misuse is a significant global health concern. In the UK, the prevalence of substance misuse has increased over the past decade and the number of alcohol and drug related deaths are increasing. Individuals with substance dependency issues are entitled to access treatment services. However, the COVID-19 pandemic created significant challenges for public services, including drug and alcohol treatment, and resulted in significant service reconfiguration and a shift from in-person to remote delivery. This study aims to evaluate the delivery of drug and alcohol services in a large metropolitan area in Northern England during the COVID-19 pandemic. It aims to understand the impact of service reconfiguration for services, staff and service users, and to use this understanding to inform the future optimised design of services.

**Design and methods:**

The study has five workstreams within a mixed methods framework: (1) Systematic review of literature; (2) Qualitative process evaluation with service providers (digital timelines, focus groups and interviews); (3) Qualitative process evaluation with service users (interviews, focus groups, text based conversations and case studies); (4) Quantitative outcomes and health economic analysis; and (5) Data synthesis and dissemination.

**Expected Impact of the study for Public Health:**

The breadth of the study, its novel nature, and the importance of substance misuse as a public health issue, mean that this study will provide valuable findings for those who commission, deliver and use drug and alcohol treatment services nationally and internationally. There will also be important learning for the effective remote delivery of services in sectors beyond drug and alcohol treatment.

## Background

### Substance misuse: The scale of the issue

The use of alcohol and illicit drugs significantly impacts the health of populations worldwide. The annual United Nations World Drugs Report found that drug use had led to more than half a million deaths in the past year^1^ and the World Health Organisation calculates that in 2016 there were three million deaths globally as a result of harmful alcohol use.^2^

In the UK, drug and alcohol misuse constitutes a significant and increasing UK public health concern. In England in 2018/19, the most recent year for which data are available, there were over 358,000 hospital admissions primarily attributed to drinking alcohol, a rise of 6% from the previous year and 19% higher than a decade before.^3^ In 2020, there were,^8^ 974 ‘alcohol-specific’ deaths (deaths categorised as a direct consequence of misusing alcohol), an 18.6% increase compared with 2019 and the highest year-on-year increase since collation of the data began.^4^

The most recent surveys of drug use in England and Wales, found that the prevalence of illicit drug use in the year to March 2020 was similar to the previous year,^5^ when it had reached its highest level in a decade. Drug mortality rates are on an upwards trajectory, with 2830 deaths from drug misuse registered in England in 2020, the highest level since records began.^6^

### Tackling substance misuse – Policy responses

The United Nations and World Health Organisation have highlighted the significant health, social and economic impacts of harmful alcohol and illicit drug use and proposed strategies and policies for addressing them.^1,2^

A recent estimate placed the social and economic costs of alcohol related harm in England at £21.5 billion and the cost of harm from illicit drug use at £10.7 billion.^7^ Significant recent UK Government policy efforts to address drug and alcohol dependency include: two recent independent reviews of drugs led by Dame Carol Black;^8,9^ the announcement of a new cross-government unit (‘Joint Combating Drugs Unit’); and a new 10-year drugs plan introduced in late 2021.^10^

### Treatment for substance dependency

The International Statistical Classification of Diseases and Related Health Problems (ICD) describes the key features of substance dependency as a strong internal drive to use a psychoactive substance, demonstrated through an impaired ability to control use, and an increased priority to use the substance despite harm or negative consequences. Those with substance dependence may also have physiological features of dependence such as withdrawal symptoms or development of tolerance to the effects of the substance.^11^

In the UK, people with substance dependency issues are entitled to access treatment that is free at the point of delivery. A common approach to services, in line with guidance,^12^ involves delivering person-centred, holistic treatment. Such treatment takes account of the wider context and system in which drug and alcohol dependency occur and are experienced, and the often multiple physical, psychological, and social needs of those with dependency issues. Approximately 20% of adults entering treatment have problems with housing and more than 50% have a mental health treatment need.^6^

The National Drug Treatment Monitoring System (NDTMS)^13^ collates UK-wide data from providers of drug and alcohol treatment services and uses four categories to describe the substance use of people in treatment: opiate; non-opiate; non-opiates and alcohol; and alcohol only. Between April 2020 and March 2021, 275,896 adults were in contact with drug and alcohol services in the UK. Over half (51%) were receiving treatment for opiate use, 28% for alcohol use only, 11% for non-opiate and alcohol use, and 9% for use of non-opiates only. However, an estimated 82% of adults in need of treatment for alcohol dependency are not in treatment. The corresponding ‘not in treatment’ figures for opiate users and crack cocaine users are 58% and 47%, respectively.^6^

### Substance misuse treatment services – The COVID-19 context

COVID-19 created significant challenges for delivering public services, including substance use treatment services.^14^ Restrictions on face-to-face meetings, social distancing, closure of some venues and services, and limitations on the numbers able to access buildings from which services are delivered, necessitated significant reconfiguration of services from March 2020 onwards. Substantial innovation in service design and delivery resulted from the need to respond to the challenges of delivering services during the pandemic.^15^

### The current study

This study aims to evaluate the delivery of drug and alcohol treatment services in a large metropolitan area in Northern England during the COVID-19 pandemic. A partnership organisation (henceforth referred to as the ‘lead organisation’) comprising National Health Service (NHS) and third sector organisations is commissioned to deliver drug and alcohol services locally on behalf of the local authority. It works in partnership with other local organisations, including charities, general practitioners (GPs), NHS trusts, pharmacies, and local authority departments. Treatment services offered include information, advice and preventative services; harm reduction initiatives; health screening; one-to-one intervention from a support worker; opioid substitution treatment; psychosocial interventions; detox; mutual aid sessions; and relapse prevention support.

COVID-19 resulted in the organisation significantly reconfiguring service delivery, involving the modification of many services, and the cessation, or scaling back, of others. Some core treatment components were moved from in-person delivery to remote delivery (delivering interventions that had previously been in-person via telephone or video platforms, for example). Prior to COVID-19, in-person delivery had been the main mode through which service users experienced drug and alcohol treatment services.

### Study aims and research questions

This study aims to understand the impact that the required changes to drug and alcohol services had on services, staff and service users, and to use this understanding to inform the optimised design of services in the future. The study’s objectives are to:

1. Identify and critically appraise the extant evidence on remote delivery of support for alcohol and/or substance use issues and harm reduction and recovery interventions for adults;
2. Investigate how drug and alcohol support services have been impacted by COVID-19 restrictions and how services were delivered during the pandemic;
3. Explore staff and service user experiences of the delivery of drug and alcohol services during the COVID-19 pandemic;
4. Assess how outcomes for service users during the COVID-19 pandemic compare with pre-COVID outcomes;
5. Explore the resource changes and associated economic impacts on the service and its service users over the pandemic;
6. Generate recommendations for how the design of drug and alcohol services might be optimised in future, drawing on lessons learnt during COVID-19;
7. Communicate the findings of the evaluation to a range of appropriate stakeholders, including service users and providers, commissioners and policymakers.

## Methods and analysis

### Study design overview

The study has five workstreams within a mixed methods framework. Each workstream addresses one or more study objectives. Table 1 maps the five workstreams to study objectives.

**Table 1.** Study workstreams mapped to objectives.

Objective	Workstreams				
	1	2	3	4	5
1. Identify and critically appraise the extant evidence on remote delivery of support for alcohol and/or substance use issues and harm reduction and recovery interventions for adults	X				
2. Investigate how drug and alcohol support services have been impacted by COVID-19 restrictions and how services were delivered during the pandemic		X	X	X	
3. Explore staff and service user experiences of the delivery of drug and alcohol services during the COVID-19 pandemic		X	X		
4. Assess how outcomes for service users during the COVID-19 pandemic compare with pre-COVID outcomes				X	
5. Explore the resource changes and associated economic impacts on the service and its service users over the pandemic		X	X	X	
6. Generate recommendations for how the design of drug and alcohol services in the area under study might be optimised in future, drawing on lessons learnt during COVID-19					X
7. Communicate the findings of the evaluation to a range of appropriate stakeholders, including service users and providers, commissioners and policymakers					X

Study inclusion criteria are that participants must be an adult age 18 or above, have the capacity to consent to participate, and have provided informed consent to participate.

Service user participants must be current or past users of drug and alcohol services within the locality under study. Staff participants must have been involved in service providers’ delivery of drug and alcohol treatment services between the start of formal COVID-19 restrictions in March 2020 and their date of recruitment into the study.

Exclusion criteria for service user participants will be individuals who: are under the age of 18 years; are not current or past users of drug and alcohol services within the locality under study; do not have the capacity to consent to participate; have not provided informed consent to participate. Service users will be excluded where they appear intoxicated at the point at which data collection is due to take place.

## Workstream 1: Literature review and existing evidence synthesis

To frame and contextualise project findings, Workstream 1 will involve conducting a systematic review of published evidence on remote delivery of drug and alcohol interventions. Full details of the questions addressed by the review, PICO and search strategy can be accessed in the review’s published Prospero Record.^16^

## Workstream 2: Qualitative process evaluation with service providers

### Design

Workstream 2 will involve qualitative data collection from staff and volunteers working for the service provider (for brevity, staff and volunteers are henceforth referred to collectively as ‘staff’) and will gather data about how service delivery changed due to and during the COVID-19 pandemic, and staff experiences of these changes.

Service providers tailor treatment depending on service users’ individual needs and circumstances, and their substance use category, using treatment ‘pathways’ that align with the four NDTMS substance categories. This workstream will utilise these pathways to frame the recruitment and sampling of participants and ensure that our sample includes representation from staff working across pathways and different areas of drug and alcohol support.

To capture the breadth and complexity of service delivery change, we will adopt a process evaluation methodology that utilises three different qualitative data collection methods: individual digital timelines, focus groups, and individual interviews. Participants will be invited to participate in at least one, and up to three of these methods. Focus groups and interviews have both been included for workstreams 2 and 3 to combine the benefits of focus groups (e.g. the ability to capture additional perspectives and depth through the interaction of participants)^17^ with those of individual interviews (e.g. allowing participants to discuss personal experiences and issues they might be reluctant to disclose in a group setting). In addition, by including both data collection methods, we hope to increase participants’ choice of available method and opportunity to participate in the research. Further detail on participant recruitment, methods used, and topics explored within each method is presented in Table 2.

**Table 2.** Further details of methods and the topics explored using each method.

Workstream 2 (staff)		
Data collection method	Further details of method	Topics explored
Individual digital timelines	Participants will be emailed a link to an online portal with an interactive, individual timeline template. The timeline will include date prompts related to key pre- and post- pandemic events. Participants will be asked to generate a timeline by placing virtual ‘post-it notes’ on the timeline to show key changes in service delivery before, during and after the implementation of COVID-19 restrictions. Participants will also be requested to indicate their experiences of these changes. Participants will be able to return to their individual timeline, which will not be able to be viewed by anyone outside the research team, at any point over a period of approximately 10 days. This will enable participants to make additions and amendments to the timeline over time and allow them to draw on prompts (e.g. notes and communications) that may aid the recall of service changes and experiences.	Topics explored will include: • how and when service delivery changed as a result of the pandemic • key service changes before, during and after the implementation of COVID-19 restrictions • participants’ experiences of service delivery changes and their perspectives on the experiences of service users
Focus Groups	Focus groups will be conducted by research team members via a video platform such as Teams or Zoom. They will last between 50 and 75 min and will be audio and video recorded. Each focus group will include staff members who contribute to a different substance use pathway, with one focus group conducted per pathway. There will be between six and eight participants per focus group (a total of approximately 28 participants).	The interview schedule will be informed by findings from individual digital timeline data collection. Topics explored will include: • how services were delivered prior to the pandemic • when and how services changed and were reconfigured in relation to particular substance use pathways • how remote modes of service delivery were implemented • how participants experienced changes in service delivery • staff perspectives on the effectiveness of service reconfiguration • any key learning for future delivery of local drug and alcohol services
Individual interviews	Individual one-to-one interviews will be conducted by research team members via a video platform such as Teams or Zoom. They will last for approximately 60 min and will be audio and video recorded. Three or four individual interviews will be conducted per substance use pathway (approximately 15 in total).	Interviews will allow the opportunity for staff to discuss issues which they may not feel comfortable broaching within a focus group context. The interview schedule will be informed by findings from the previous data collection activities. Topics explored will include: • participants’ experiences of changes in service delivery • participants’ perspectives on service users’ experiences of changes in service delivery • ways in which individual contexts and circumstances have contributed to and influenced the experience of change • what has worked well and less well in terms of remote delivery • impacts of service reconfiguration on service provider-service user relationships • the nature of participants’ relationships with their organisation and whether and how these have changed • any key learning for future delivery of local drug and alcohol services
Workstream 3 (service users)		
Data collection method	Further details of method	Topics explored
Interviews	Individual one-to-one interviews will be conducted by research team members via telephone or a video platform (such as Teams or Zoom), according to participant preference. We will endeavour to work with local digital inclusion organisations to enable participation from those without easy access to tablets, smartphones, wi-fi, or adequate private space. Examples of such organisations include a local initiative that is able to loan digital devices such as tablets. Where COVID-19 restrictions allow, we will also work with drug and alcohol service providers to facilitate wi-fi access via their buildings, for participants who lack such access but would like to participate in the study. Interviews will last for approximately 60 min and will be audio and video recorded.	Interviews will aim to provide insight into how service users’ substance use category, treatment status and other demographic and contextual factors influenced their experience of services. Topics explored will include: • participants’ experiences of service delivery changes (including how they were informed about changes) • participants’ changing experiences of services during the pandemic • what has worked well and less well for service users in terms of remote delivery and other aspects of service reconfiguration • barriers to engagement with treatment services • service users’ suggestions for how services could best be configured in future to meet their needs
Focus groups	Focus groups will be conducted by research team members via a video platform such as Teams or Zoom and will last between 50 and 75 min. They will be audio and video recorded. To encourage and facilitate participation, participants will have the option of taking part with their cameras off and/or accessing the focus group by dialling in using a telephone.	Topics explored will include: • participants’ experiences of service delivery changes (including how they were informed about changes) • participants’ changing experiences of services during the pandemic • what has worked well and less well for service users in terms of remote delivery and other aspects of service reconfiguration • barriers to engagement with treatment services • service users’ suggestions for how services could best be configured in future to meet their needs
Text based conversations	Participants will be invited to engage in an asynchronous text-based conversation (e.g. via WhatsApp, SMS text-message, or email). The platform on which the discussion takes place will depend on the preference of the service user. Conversation will commence with a research team member asking the participant an initial question from a pre-prepared schedule of questions. Participants will be encouraged to respond in their preferred timescale and the research team member will then aim to respond within a few hours of obtaining that response. The aim will be to generate a free-flowing discussion between researcher and participant, exploring topics similar to those explored using the interview method above. We will aim to limit the degree of invasiveness into the lives of participants and therefore if participants do not reply to a communication from the research team within 48 h, no more than two subsequent prompts will be sent.	Topics explored will include: • participants’ experiences of service delivery changes (including how they were informed about changes) • participants’ changing experiences of services during the pandemic • what has worked well and less well for service users in terms of remote delivery and other aspects of service reconfiguration • barriers to engagement with treatment services • service users’ suggestions for how services could best be configured in future to meet their needs

## Recruitment and sampling

Participants will be recruited from current staff members at the lead organisation and two key partner organisations (henceforth, these organisations are collectively referred to as ‘service providers’). To recruit potential participants, service providers will use internal email lists to invite staff to volunteer to participate. Those who subsequently volunteer will be asked to provide us with their basic details (e.g. work contact details and job role) via Research Electronic Data Capture (REDCap), a secure online platform.^18^ This registration process will generate a list of potential staff participants.

With registration complete, recruitment for the timeline method will begin. Maximum variation sampling^19^ will be used to obtain a sample which represents, within sample size constraints, variation in terms of staff job roles and specialities, seniority (e.g. managers and frontline staff), time in post, ‘pathway’ supported, locality, and experience of working remotely since the start of the pandemic (yes or no). Selected potential participants will then be invited to read the Participant Information Sheet (PIS) and provide e-consent via REDCap, before being invited to complete a digital timeline.

Each subsequent data collection method will begin once the preceding method has been completed, with maximum variation sampling used with each method to achieve a spread according to the criteria described above. Workstream 2 will recruit:

- Method 1 – Individual digital timelines: Approximately 25 participants
- Method 2 – Focus groups: Approximately 28 participants (across four focus groups)
- Method 3 – Individual interviews: Approximately 15 participants

The stated number of participants is that which we believe will be sufficient for data saturation to be reached. We will conduct fewer interviews if both data and meaning saturation^20^ are achieved before the stated numbers have been completed. Staff participants’ involvement in the study will not be communicated to service providers.

Further detail on the methods used and the topics explored within each is presented in Table 2.

## Workstream 3: Qualitative process evaluation with service users

### Design

Workstream 3 will utilise qualitative data collection with service users to explore their experiences of accessing support during the COVID-19 pandemic and how their experiences compare with pre-COVID. The diverse population in receipt of services necessitates a multi-faceted, inclusive research design. Our design therefore utilises three ‘core’ data collection methods: interviews, focus groups, and text-based conversations (included as they enable engagement with the study by those who might not feel able to engage with the other two more ‘traditional’ methods and can help to increase engagement in research by people from marginalised populations^21^), alongside a ‘case study’ method^22^ included to allow the perspectives of a cohort of often ‘underheard’ service users to be represented in the findings.

## Recruitment and sampling: Core data collection

Service users will be recruited with the assistance of service providers, who will be asked to disseminate study information as widely as possible among service users via a range of publicity methods. Service users interested in participating will be asked to read a PIS and complete a Consent Form and Registration Form (requesting information such as name, contact details, substance use category, demographic information, and which of the data collection methods they would be willing to participate in) via REDCap. Hard copies will be made available via service providers where their COVID-19 guidelines permit.

Where service users are unable to read the study documentation themselves or self-complete the consent form, staff will be available to assist with completion, subject to COVID-19 guidelines. Similarly, where restrictions allow, service providers will provide service users with access to IT equipment within their venues to help alleviate digital exclusion issues.

Once registered and consented, maximum variation sampling will be used to obtain a sample which represents, within sample size constraints, variation in terms of time in treatment, age, ethnicity, gender, organisation from whom support has been received, and experience of being in treatment pre and/or post the pandemic. Where a participant declines to participate when contacted or does not engage with a method, a replacement participant with similar characteristics will be selected.

This workstream will recruit:

- Interviews: 16 participants (ideally, four per substance use treatment ‘pathway’).
- Focus groups: Between 20 and 32 service users (4 focus groups, each involving between 5 and 8 participants). Participants will be grouped based on their ‘pathway’, treatment status or other demographic characteristics.
- Text-based conversations: Approximately 16 service users.

The stated number of participants is that which we believe will be sufficient for data saturation to be reached. We will conduct fewer interviews and text-based conversations if both data and meaning saturation^20^ are achieved before the stated numbers have been completed. Service users’ involvement in the study will not be communicated to service providers.

Further detail on the methods used and the topics explored using each method is presented in Table 2.

## Additional data collection: Case studies highlighting street sex workers’ pandemic experiences of treatment services

During the research planning phase, service providers highlighted that women engaged in on-street sex work are a service user group towards whom specific provision is aimed, but whose voices are typically ‘underheard’ in research and who might be particularly unlikely to engage with any of our three planned ‘core’ data collection methods. To enable the perspectives of members of this population to be reflected within the study, we will use an additional data collection method that involves working with staff who support them.

We will gather the perspectives of women engaged in on-street sex work through the trusted relationships already established between these women and their support workers. Staff will participate in research methods training facilitated by a research team member to support them in holding, during their routine support work, focussed conversations with women about their experiences of accessing treatment. Subsequently, staff will hold conversations with women and compile anonymous field notes to help them capture service users’ responses. A further workshop will then be held with staff, a research team member and a creative writing facilitator, to assist staff in translating their field notes into anonymous ‘case studies’ that highlight on-street sex workers’ experiences of treatment services during the pandemic.

## Workstreams 2 and 3: Data analysis

Text-based conversation and transcribed focus group and interview data will be coded and managed using NVivo,^23^ as will timeline data where relevant. Data will be analysed using framework analysis^24^ which is particularly suited to analysis of large qualitative datasets by multiple researchers. Case study outputs will be treated as discrete outputs rather than as raw data to be analysed. Findings will be synthesised across Workstreams 2 and 3 ahead of further synthesis in Workstream 5 (Table 2).

## Workstream 4: Quantitative outcomes and health economic analysis

This workstream will utilise pseudonymised routinely collected service delivery and service user contact data provided by the lead organisation. A data sharing agreement will be in place with the organisation prior to secure data transfer. Pseudonymised data for the 12 months preceding and the 12 months following the start of COVID-19 restrictions will be analysed, with the sample size determined by the number of records available, but likely to exceed 3000.

## Quantitative outcomes evaluation

Analysis will aim to assess how outcomes for service users during the COVID-19 pandemic compare with pre-COVID outcomes. The specific issues examined by the analysis will be:

1. Changes in the mix of service user ‘treatment stage’, with relatively fewer new service users indicating possible challenges to engaging those requiring treatment.
2. Changes regarding outcomes prior to and after the start of the pandemic.
3. Shifts in the relative importance of potential explanatory factors (e.g. ethnicity, age) as the pandemic evolved.

The Treatment Outcomes Profile (TOP) is the standard national outcome monitoring measure used by substance misuse services in England.^25^ It is routinely completed by those in treatment and TOP data collated by the lead organisation will be central to the outcomes analysis.

Analysis of these data will allow service user categorisation by service user ‘treatment stage’ (‘new’, ‘ongoing’ or ‘exiting’) and outcomes variables will be selected or derived from this measure. Outcomes measures based on TOP data will include substance use over a 28-day period, accommodation status, service user rated quality of life, psychological health and physical health, engagement in criminal activity, and engagement in constructive activity (e.g. work or education).

Service user variables will include age, gender, ethnic background, employment status, and Index of Multiple Deprivation (IMD) quintile. Service users within the four substance use categories will be analysed separately.

Linear regression lines will be fitted to outcome data collected each month to examine trends. A runs test on the signs generated will assess clustering of positive/negative differences.

To adjust for covariates, multivariable analysis of outcome variables will be performed at 6-month intervals between March 2019 and March 2021. This will enable comparison of pre-pandemic with medium-term post-pandemic data. For computations, Stata Version 15.1 will be used.^26^

Descriptive statistics will be used to summarise binary and categorical variables, to assess treatment stage mix, and to report service user numbers by month and substance category.

## Health economic evaluation

As with the quantitative outcomes evaluation, the health economics evaluation will utilise the routinely collected quantitative data held in the records of the lead organisation. The analysis will involve three components. Firstly, we will conduct cross-sectional analysis of service activity data to explore, on a month-by-month basis, how activities delivered by the lead organisation changed over the pandemic period. This will consider overall service delivery and delivery separated by service user substance use category.

The second component will involve conducting workshops with staff to identify a small number of key activities that are believed to have changed significantly during the pandemic and subsequently exploring how the resources and costs involved in delivering them has changed.

Aspects of service delivery likely to form part of this analysis include duration of sub-activities and staffing requirements. We anticipate that staff time will be the major resource involved in delivery and this will be costed by multiplying the duration of activities by hourly rates of employment based on staff grade. Costing will take account of overheads (e.g. national insurance contributions). Other resources that have been impacted (e.g. room hire) will also be assessed and costed where feasible.

The third component will involve integrating findings from Workstream 2 and 3 about out-of-pocket expenses incurred by service users (e.g. costs related to travel or internet access) and summarising how these varied with the move towards remote delivery of services to explore implications for service access and equity.

## Workstream 5: Data synthesis and dissemination

This workstream will synthesise the findings from Workstreams 1 to 4 and develop recommendations about how to configure and deliver services in the locality under study in the future. Qualitative and quantitative data will primarily be integrated at the ‘interpretation and reporting’ level^27^ and a contiguous/weaving approach will be adopted.^28^

Recommendations will be generated through broad consultation (e.g. with the project Advisory Group, service providers, and service users) and further developed with key drug and alcohol service and sector stakeholders at workshops held after completion of data analysis.

Our impact, implementation and dissemination work will involve working closely with a broad range of stakeholders to understand how findings can be most effectively communicated and mobilised, and we anticipate disseminating findings through multiple routes (e.g. academic journal articles, blog posts, briefing notes, social media, and summary reports).

## Patient and public involvement (PPI)

A PPI group, known as the Public Involvement in Research group (PIRg), is an integral part of the research process and will provide PPI input into this evaluation. The PIRg have advised on key aspects of our methodology, including the literature review and data collection methods, and will continue to support data analysis, dissemination, and implementation work. Their input will be complemented by local service users with lived experience of accessing drug and alcohol support services. A group has been convened for this purpose, with the assistance of the lead organisation. PIRg members and local services users involved in PPI work will be remunerated in line with NIHR guidance.^29^

## Discussion and expected impact for Public Health

This study aims to evaluate the redesign of local drug and alcohol services necessitated by the COVID-19 pandemic. Our mixed contiguous/weaving approach to analysis will integrate findings from workstreams 1, 2, 3 and 4 to produce a comprehensive and multi-faceted description of staff and service users’ experiences of service delivery, the structure and delivery of drug and alcohol services prior to and since the beginning of the COVID-19 pandemic, and the implications of service reconfiguration and remote delivery for service users, staff and organisations.^26^

The study will generate learning that is relevant to the drug and alcohol sector and beyond, by co-producing recommendations for practice through an iterative process of sharing and refining ‘key messages’ from the research. This process will be led by the research team and involve consultation with drug and alcohol service stakeholders (including service users and staff), the Advisory Board, PIRg, and project-specific Advisory Group. It will culminate in a series of stakeholder workshops, during which recommendations for future optimisation of drug and alcohol services will be further developed and refined to ensure they are appropriate and feasible.

To ensure that the findings of the study can inform practice, our knowledge mobilisation, implementation, and impact work will consider factors such as: the value of findings to the drug and alcohol sector and wider public health system; what outputs would be most valuable to particular audiences; and how outputs might be most effectively communicated and mobilised. The nature and format of outputs will be decided in close consultation with project stakeholders and drug and alcohol sector experts to ensure that study findings achieve maximum impact.

Particular focus will be placed on assessing what works well, less well, for whom and in what circumstances, and on providing recommendations for the possible implementation of a future ‘hybrid’ form of service delivery that combines remote and in-person delivery. Ensuring that the reconfiguration of services does not exacerbate existing health inequalities, necessitates consideration of the differential needs and circumstances of service users, including disparities in levels of digital inclusion. The study will provide valuable learning about barriers to service users’ access to digital delivery, how these might be addressed, and how digital modes of drug and alcohol service delivery can be equitably implemented.

Key strengths of the study design are the multiple perspectives it will capture (service users, staff and organisations) and the mixed method approach adopted. The study’s novel nature (few studies of remote delivery of drug and alcohol services during the pandemic have thus far been conducted), and the importance of substance misuse as a public health issue, mean that the study will provide valuable findings for those who commission, deliver and use drug and alcohol treatment services nationally and internationally. There will also be important learning for the effective remote delivery of services in sectors beyond drug and alcohol treatment.

## References

[1] United Nations Office on Drugs and Crime. World drug report 2021. UNODC, https://www.unodc.org/unodc/en/data-and-analysis/wdr2021.html (June 2021, accessed 12 November 2021).

[2] World Health Organization. Global status report on alcohol and health 2018. World Health Organization, https://www.who.int/substance_abuse/publications/global_alcohol_report/en/ (September 2018, accessed 1 November 2021).

[3] NHS Digital. Statistics on alcohol, England 2020. NHS Digital, https://digital.nhs.uk/data-and-information/publications/statistical/statistics-on-alcohol/2020 (February 2020, accessed 1 December 2021).

[4] Office for National Statistics. Alcohol-specific deaths in the UK: registered in 2020, Statistical bulletin, release date December 7, 2021. Office for National Statistics, https://www.ons.gov.uk/peoplepopulationandcommunity/healthandsocialcare/causesofdeath/bulletins/alcoholrelateddeathsintheunitedkingdom/registeredin2020 (December 2021, accessed 15 December 2021).

[5] Office for National Statistics. Drug misuse in England and Wales: year ending March 2020. Office for National Statistics, https://www.ons.gov.uk/peoplepopulationandcommunity/crimeandjustice/articles/drugmisuseinenglandandwales/yearendingmarch2020 (2020, accessed 25 November 2021).

[6] Office for Health Improvement and Disparities. Adult substance misuse treatment statistics 2020 to 2021: report. Office for Health Improvement and Disparities, https://www.gov.uk/government/statistics/substance-misuse-treatment-for-adults-statistics-2020-to-2021/adult-substance-misuse-treatment-statistics-2020-to-2021-report (November 2021, accessed 1 December 2021).

[7] Public Health England Alcohol and Drugs Prevention. Treatment and recovery: why invest? Public Health England, https://www.gov.uk/government/publications/alcohol-and-drug-prevention-treatment-and-recovery-why-invest/alcohol-and-drug-prevention-treatment-and-recovery-why-invest (February 2018, accessed 3 November 2021).

[8] Department of Health and Social Care. Review of drugs: phase one report. Department of Health and Social Care, https://www.gov.uk/government/publications/review-of-drugs-phase-one-report (February 2020, accessed 15 September 2021).

[9] Department of Health and Social Care. Review of drugs: phase two report. Department of Health and Social Care, www.gov.uk/government/publications/review-of-drugs-phase-two-report (August 2021, accessed 3 November 2021).

[10] HM Government. From harm to hope: a 10-year drugs plan to cut crime and save lives. HM Government, https://assets.publishing.service.gov.uk/government/uploads/system/uploads/attachment_data/file/1039240/From_harm_to_hope_PDF-final_bookmarked_v3.pdf (December 2021, accessed 15 December 2021). 10.1016/j.drugpo.2022.103840 36068144

[11] World Health Organization. ICD-11: International Statistical Classification of Diseases and Related Health Problems (ICD) 11th Revision. World Health Organization, https://www.who.int/standards/classifications/classification-of-diseases (2019, accessed 17 February 2022).

[12] Clinical Guidelines on Drug Misuse and Dependence Update 2017 Independent Expert Working Group. Drug misuse and dependence: UK guidelines on clinical management 2017. Department of Health, https://www.gov.uk/government/uploads/system/uploads/attachment_data/file/628634/clinical_guidelines_2017.pdf (July 2017, accessed 15 November 2021).

[13] NHS Digital. National drug treatment monitoring system, https://digital.nhs.uk/data-and-information/information-standards/information-standards-and-data-collections-including-extractions/publications-and-notifications/standards-and-collections/dcb0107-national-drug-treatment-monitoring-system (2021, accessed 15 December 2021).

[14] Scott J Millar J Family H , et al. What C-OST? Impact of the COVID-19 pandemic on people who receive opioid substitution therapy in rural areas. Interim report – number 2, https://arc-w.nihr.ac.uk/Wordpress/wp-content/uploads/2021/06/What-Cost-Second-Insights-Report-May-2021-1.pdf (May 2021, accessed 28 November 2021).

[15] House of Lords Public Services Committee. A critical juncture for public services: lessons from COVID 19, 1st report of session 2019-21. November 2020. HL Paper 167.

[16] Garcia Iglesias J Howlett N Breslin G , et al. Remote delivery of alcohol and/or substance use disorder interventions for adults: a systematic review. PROSPERO 2021 CRD42021234116, https://www.crd.york.ac.uk/prospero/display_record.php?ID=CRD42021234116 (January 2021, accessed 25 November 2021). 10.1371/journal.pone.0259525 PMC8562816 34727134

[17] Lambert SD Loiselle CG . Combining individual interviews and focus groups to enhance data richness. J Adv Nurs 2008; 62(2): 228–237. 18394035 10.1111/j.1365-2648.2007.04559.x

[18] Harris PA Taylor R Thielke R , et al. Research electronic data capture (REDCap)–a metadata-driven methodology and workflow process for providing translational research informatics support. J Biomed Inform 2009; 42(2): 377–381. 18929686 10.1016/j.jbi.2008.08.010 PMC2700030

[19] Patton M . Qualitative evaluation and research methods. Beverly Hills, CA: SAGE, 1990.

[20] Hennink MM Kaiser BN Marconi VC . Code saturation versus meaning saturation: how many interviews are enough? Qual Health Res 2017; 27(4): 591–608. 27670770 10.1177/1049732316665344 PMC9359070

[21] Spencer T Rademaker L Williams P , et al. Asynchronous data collection in qualitative research. In: Leavy P (ed.) The oxford handbook of methods for public scholarship. Oxford: Oxford University Press, 2019, pp.443–468.

[22] Yin RK . Case study research: design and method. New York, NY: SAGE Publications, 1994.

[23] QSR International Pty Ltd. NVivo. Released in March 2020, https://www.qsrinternational.com/nvivo-qualitative-data-analysis-software/home (accessed 12 December 2021).

[24] Ritchie J Spencer L . Qualitative data analysis for applied policy research. In: Bryman A Burgess RG (eds) Analyzing qualitative data. London: Routledge, 1994, pp.173–194.

[25] Marsden J Farrell M Bradbury C , et al. Development of the treatment outcomes profile. Addiction 2008; 103(9): 1450–1460. 18783500 10.1111/j.1360-0443.2008.02284.x

[26] Stata Corp LLC. Stata statistical software, Release 15. College Station, TX: StataCorp LLC, 2017.

[27] Fetters MD Curry LA Creswell JW . Achieving integration in mixed methods designs-principles and practices. Health Serv Res 2013; 48(6, Pt 2): 2134–2156. 24279835 10.1111/1475-6773.12117 PMC4097839

[28] Fetters MD Freshwater D . Publishing a methodological mixed methods research article. J Mix Methods Res 2015; 9(3): 203–213.

[29] National Institute of Health. Research Payment guidance for members of the public considering involvement in research. National Institute of Health, April 2021, https://www.nihr.ac.uk/documents/payment-guidance-for-members-of-the-public-considering-involvement-in-research/27372

